# Evaluation of the Effects of Crushed and Expanded Waste Glass Aggregates on the Material Properties of Lightweight Concrete Using Image-Based Approaches

**DOI:** 10.3390/ma10121354

**Published:** 2017-11-25

**Authors:** Sang-Yeop Chung, Mohamed Abd Elrahman, Pawel Sikora, Teresa Rucinska, Elzbieta Horszczaruk, Dietmar Stephan

**Affiliations:** 1 Building Materials and Construction Chemistry, Technische Universität Berlin, Gustav-Meyer-Allee 25, 13355 Berlin, Germany; sychung419@gmail.com (S.-Y.C.); abdelrahman@tu-berlin.de (M.A.E.); stephan@tu-berlin.de (D.S.); 2 Structural Engineering Department, Mansoura University, Elgomhouria St., Mansoura City 35516, Egypt; 3 Faculty of Civil Engineering and Architecture, West Pomeranian University of Technology Szczecin, Al. Piastow 50, Szczecin 70-311, Poland; trucinska@zut.edu.pl (T.R.); elzbieta.horszczaruk@zut.edu.pl (E.H.)

**Keywords:** crushed waste glass, expanded waste glass, recycling, lightweight concrete, thermal conductivity, compressive strength

## Abstract

Recently, the recycling of waste glass has become a worldwide issue in the reduction of waste and energy consumption. Waste glass can be utilized in construction materials, and understanding its effects on material properties is crucial in developing advanced materials. In this study, recycled crushed and expanded glasses are used as lightweight aggregates for concrete, and their relation to the material characteristics and properties is investigated using several approaches. Lightweight concrete specimens containing only crushed and expanded waste glass as fine aggregates are produced, and their pore and structural characteristics are examined using image-based methods, such as scanning electron microscopy (SEM), X-ray computed tomography (CT), and automated image analysis (RapidAir). The thermal properties of the materials are measured using both Hot Disk and ISOMET devices to enhance measurement accuracy. Mechanical properties are also evaluated, and the correlation between material characteristics and properties is evaluated. As a control group, a concrete specimen with natural fine sand is prepared, and its characteristics are compared with those of the specimens containing crushed and expanded waste glass aggregates. The obtained results support the usability of crushed and expanded waste glass aggregates as alternative lightweight aggregates.

## 1. Introduction

These days, there are many concerns related to industrial by-products because of their harmful effects on the environment and energy consumption. Many efforts have been undertaken to find a way to reduce these negative effects and to enhance sustainability in technical and economic terms [1]. The recycling of waste materials has been considered as an approach for reducing the amount of discarded materials and thus dealing with the by-product problem. In the building and civil engineering fields, the amount of waste material is increasing continuously with developments in the construction industry. Building materials, such as concrete, are the most widely produced materials in the world, and the utilization of waste materials as concrete ingredients is being strongly promoted [2]. In the last few decades, the use of various by-products and waste materials has become an established and fast-growing industry [3].

Solid waste materials can be utilized as both powdered material and as aggregates for concrete. The use of waste materials from construction and demolition as aggregates is considered to be a promising approach due to a shortage in natural aggregates (which constitute 60–80 % concrete by volume) and the resultant reduction in the relatively large energy consumption associated with the processing of raw constituents [4]. For instance, cement kiln dust [5], municipal solid waste incinerator 34 (MSWI) fly ash [6], and non-metallic shredder [7] can effectively produced and used as lightweight aggregates with high economical and environmental impacts. Glass is also one of the most commonly used materials in various industries, and large amounts of waste glass (WG) are generated in many countries; therefore, concrete which utilizes waste glass can have many positive effects from an environmental and engineering perspective. In particular, WG can be effectively used as an ingredient in cement-based composites since the chemical composition of WG is similar to that of natural sand (i.e., all commercial glass products contain more than 70 % of SiO${}_{2}$). Theoretically, WG can be recycled completely and infinitely without loss in its chemical and physical properties [8].

At the same time, the use of WG has some disadvantages because of its broken, mixed colored, and diverse origin of waste glass which cause impractical and highly expensive in the recycling process [9,10,11]. As a result, WG is currently mainly used as a fine waste glass fraction which cannot be processed for further recycling or is dumped in landfills. To overcome these limitations, several studies related to the utilization of WG as an aggregate and powder have been performed. For example, WG has been adopted as a construction material in paving blocks [3], road application (so-called glasphalts) [12], shielding cement mortar [13], screeds [14], architectural mortars [15], high performance concrete [16], self-cleaning concrete [17] and bactericidal mortars [18]. WG aggregates were also utilized to prepare alkali-activated materials such as blast furnace slag or fly ash [19] and alkali-activated concretes [20]. In addition, the alkali-silica reaction (ASR) should be carefully considered for the use of WG aggregate in cement-based composites due to its chemical characteristics. In concrete materials, amorphous silica can be dissolved in glass under alkaline conditions to form an ASR gel, which can cause expansion and cracking [21]. Previous researches showed that mortars made of green and brown glass sands are innoxuous, while clear glass can have deleterious properties [22,23]. The same result was reported in [24] that all cement mortars containing soda lime-glass met the permissible limits at satisfactory level. Nevertheless, many researchers have demonstrated that ASR can be effectively controlled by optimizing the mix composition, through the use of supplementary cementitious materials, such as fly ash, ground granulated blast furnace slag, metakaolin, silica fume or even waste glass powder [17,23,25,26].

WG aggregates have been used to enhance the specific material property of concrete materials. For instance, Yu et al. [11] have used an expanded-glass lightweight aggregate for lightweight concretes to improve their thermal properties. Several studies have shown that partial replacement (up to 40 vol %) of natural fine aggregate with WG fine aggregate has had a beneficial effect on the mechanical properties of cement mortars and concretes [18,23,25,27]. In particular, Serifou et al. [28] has demonstrated that the incorporation of coarse WG aggregate can lead to a deterioration of the mechanical properties of cement-based composites due to the cracking potential of the WG coarse aggregate; therefore, the utilization of fine WG aggregates along with natural coarse aggregates can be more effective in the incorporation of cementitious-composites. Nevertheless, entirely substituting natural fine aggregates with WG fine aggregate is more desirable than partial replacement from environmental, economic, and sustainability perspectives. Alani et al. [14] has demonstrated that the substitution of natural fine aggregate with WG fine aggregate significantly decreases the thermal conductivity of screeds (from 2.0 to 0.7 W/m/K), and Guo et al. [15] has shown that a particularly high ratio of natural aggregate with WG replacement can decrease the thermal conductivity of cement mortars. Krishnamoorthy et al. [29] have also shown that the partial replacement of fine aggregate with WG fine aggregate (from 10 to 50 vol %) in concrete can gradually decrease thermal conductivity, as the recycled glass content of a specimen increases.

Modern building materials should meet minimum mechanical property and durability requirements so as to be successfully used in construction fields. Furthermore, it has been suggested that energy savings can be made through the proper insulation of a structure; therefore, thermal conductivity is becoming an important factor in determining the properties of modern building materials [30]. This study is aimed to evaluate the effect of crushed and expanded WG in production of lightweight concretes and their effect on thermal and mechanical properties. As a control sample concrete containig naturals sand was prepared. The main objectives of this study are summarized as follows: (1) Production of lightweight concrete specimens with crushed and expanded WG aggregates, respectively, by replacing all fine aggregates with WG aggregates; (2) Application of a grading curve to maximize the volume of the crushed and expanded WG aggregates inside the specimens; (3) Investigation of the effects of crushed and expanded WG aggregates on pore characteristics (via image-based techniques) and the thermal and mechanical properties of the materials. For these purposes, lightweight concrete specimens that contained only crushed and expanded WG aggregates (100%) as fine aggregates were prepared. For all aggregate types, the same grading curve [31] was adopted. To investigate microstructural characteristics, such as pore and solid structures, scanning electron microscopy (SEM), X-ray computed tomography (CT), and other image-based methods (RapidAir), were utilized [32,33,34]. The correlation between the characteristics and properties of the specimens with crushed and expanded WG aggregates was investigated, and the effects of different aggregate types on the materials was also examined. Based on the results obtained, the utilization of crushed and expanded WG as alternative aggregates is discussed below.

## 2. Preparation of Samples with Different Aggregates

### 2.1. Materials

**Figure 1:**
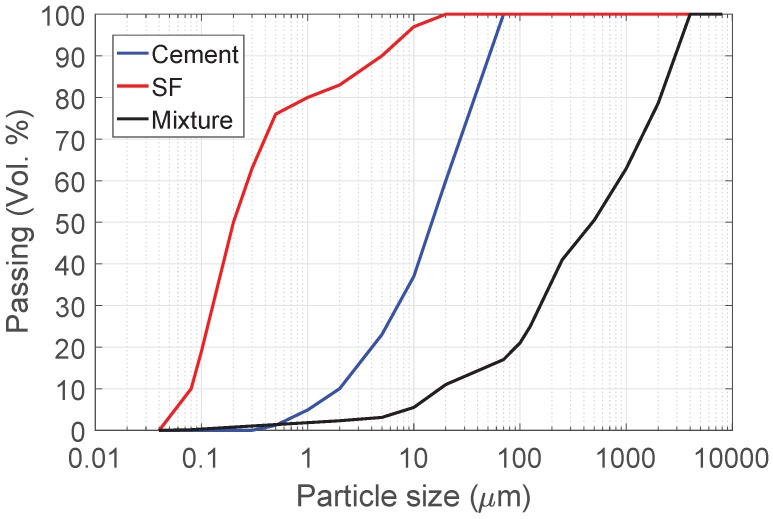
Particle size distributions of cement, silica fume (SF), and the concrete mixture.

Ordinary Portland cement CEM I 42.5 N according to DIN EN 197-1 provided by HeidelbergCement (Leimen, Germany) and condensed silica fume according to DIN EN 13263-1 provided by Sika Deutschland (Leimen, Germany) were used to prepare the concrete specimens. Table 1 presents the chemical and physical compositions of cement and silica fume. The particle size distributions of cement and silica fume are given in Figure 1. Three different aggregates were used in this study: crushed waste glass, expanded waste glass (Poraver${}^{®}$, Dennert Poraver GmbH, Schlüsselfeld, Germany), and natural sand with a maximum aggregate size of 4 mm. The physical properties of the used aggregates can be found in Table 2. In order to eliminate the effect of aggregate grading on the properties of the concrete, all three aggregates were sieved and fractioned so that almost the same amount of each fraction (by volume) was included. An ether-based polycarboxylic superplasticizer (Sika Viscocrete 1051, Berlin, Germany) with a density of 1.04 g/cm${}^{3}$ was used to achieve a consistency class of F4/F5, according to EN 206-1. To improve the stability of expanded glass mixes and to prevent segregation, a viscosity enhancing admixture (Sika Stabilizer, type 10160317, Berlin, Germany) was used.

Crushed glass aggregates were prepared from brown soda-lime waste glass obtained from a local recycling company; this glass is generally used for the production of beverage glass containers. Waste glass was washed with water (to remove organic contaminants), dried, and crushed in a planetary ball mill in order to obtain the desired particle size. The particle size distributions of the three aggregates used in the study were identical and are presented in Figure 1. Waste glass can be used in concrete as aggregate directly after cleaning and crushing as discussed above. In addition, it can be finely ground and expanded. Poraver${}^{®}$ is an expanded glass granulate, which is produced of recycled glass. The glass is finely ground and sintered at a temperature of 750 ${}^{\circ }$C to 900 ${}^{\circ }$C in rotary kiln. It has smooth surface with very low density (300–800 kg/m${}^{3}$) and it is composed of closed external shell and a number of internal air pores encapsulated within the shell [11]. Expanded glass has the advantages of absorbing less water than expanded clays and also to have lower thermal conductivity. It is standardized building product according to DIN EN 13055-1. Thermal conductivity of Poraver${}^{®}$ is 0.07 (W/m/K) and the compressive strength ranges between 1.4–2.8 (MPa) depending on the aggregate size.

**Figure 2:**
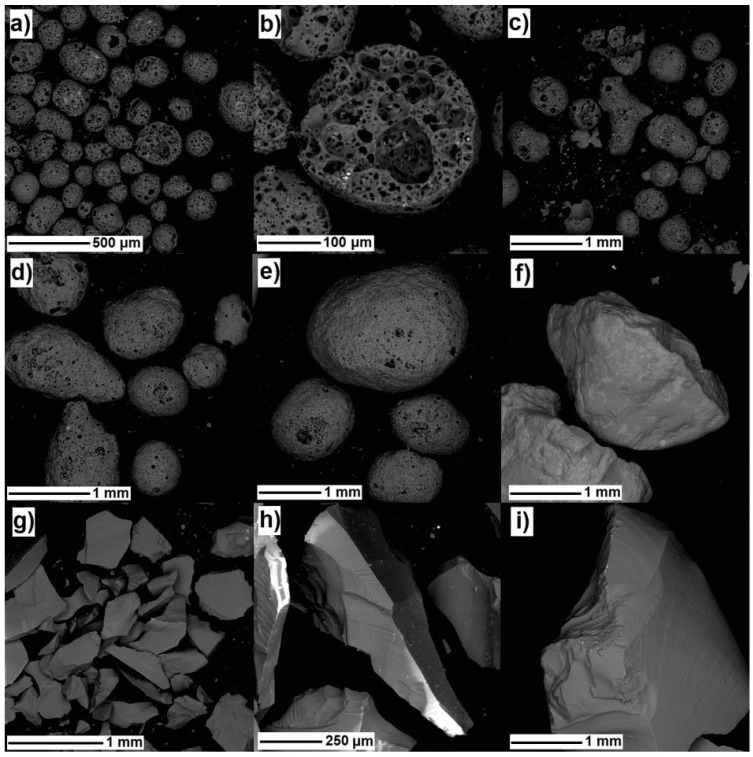
SEM micrographs of expanded glass fraction: (**a**)–(**b**) 0.125–0.250 mm; (**c**) 0.25–0.50 mm; (**d**) 0.5–1 mm; (**e**) 1–2 mm; (**f**) river sand fraction 2–4 mm; (**g**) crushed glass fraction 0.25–0.50 mm; (**h**) 0.5–1 mm; (**i**) 1–2 mm.

Scanning electron microscope (SEM) micrographs of the tested aggregates are presented in Figure 2. In general, expanded waste glass aggregate (Poraver${}^{®}$) is a highly porous material, although the porosity of the aggregate varies within its fractions. Finer fractions of expanded glass aggregates in Figure 2a–c are much more porous than the coarser particles (Figure 2d–e), and due to its high porosity, expanded glass aggregate is considered to be a brittle material (Figure 2c); therefore, the mechanical properties of composites containing this type of aggregate are relatively low. In addition, in the case of a high mixing speed, coarser particles of aggregate tend to be damaged during the mixing process, which can lead to a change in the particle size distribution of an aggregate and to an increase in the water demand of a fresh mix.

The surface of crushed glass in Figure 2g–i is different from that of the natural sand aggregate (Figure 2f). The surface of the glass particle is much smoother and more impermeable than that of natural aggregate. It can be observed that the crushed glass aggregates (Figure 2g,h) are generally angular-shaped and contain flat and elongated particles. As reported in [35], the degree of angularity as well as the amount of flat and needle-shaped particles depends on particle size and the aggregate preparation methods (i.e., the crushing method). In particular, finer fractions of crushed glass were found to consist of relatively less rough angular, flat, and elongated particles, as compared with the coarser fractions; therefore, finer crushed glass aggregate particles are more similar to that of natural aggregate which is round and smooth, even though extra crushing is required to obtain fine fractions [35].

### 2.2. Mix Proportions

In this study, three different mixes were designed and prepared. The concrete specimens with crushed waste glass, expanded waste glass, and natural sand are denoted as CG, EG, and NS, respectively. The aggregate grading of all the mixes were fixed in order to clarify the effects of aggregate type on the properties of concrete. Each aggregate was sieved separately, and the required amount of each fraction was taken to fit the grading curve of the mixture as shown in Figure 1. Moreover, the binder content was set to be 90 wt % of cement and 10 wt % of silica fume in all mixes.The w/b ratio used in this study was 0.6. The aggregates were used in a dry form and the amount of water equals to the water absorption has been added to the water needed for cement (w/b). The water absorption of aggregate is not included in the w/b and added directly during mixing. Details of concrete mix proportioning are given in Table 3. A bucket mixer was used to mix the concrete constituents. Superplasticizer was added, and the consistency of fresh concrete was measured according to EN 12350-5 using a flow table test. The flow diameter of all mixes was in the range of 550–600 mm. After measuring the properties of fresh concrete, 10 cm × 10 cm × 10 cm and 15 cm × 15 cm × 15 cm cubical molds were cast for measuring compressive strength and thermal conductivity, respectively. Moreover, standard prisms 4 cm × 4 cm × 16 cm were prepared from each mix to measure flexural strength. In each test, six samples were tested and the mean value was considered. 24 h after concreting, the test specimens were demolded and cured under water at room temperature (20 ± 1 ${}^{\circ }$C) until testing.

## 3. Characterization and Property Evaluation

### 3.1. Porosity Measurements

**Figure 3:**
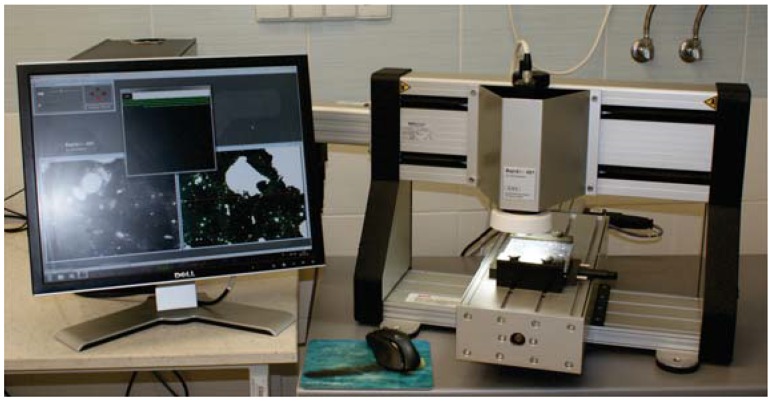
RapidAir 457 device to analyze the pore characteristics.

In this study, image based techniques, such as X-ray computed tomography (CT) and automated image analysis systems based on a linear traverse method, were incorporated to investigate microstructural characteristics, such as pore and solid structures. The results were supported with Tescan Vega 3 scanning electron microscope (SEM) microstructure analysis. A characterization of the air-void structure of hardened concrete, using a linear traverse method (EN 480-11) was performed automatically with a RapidAir 457 Automated-Air-Void-Analyzer (Concrete Experts International, Sweden), as reported in Figure 3. The RapidAir 457 consists of a computerized control unit (PC) with a color monitor, video camera, and microscope objective mounted on a moving stage. For testing, cured concrete samples were cut into 1 cm-thick slices with a size of 15 cm × 15 cm at the middle of the specimen. After cutting the samples with a diamond blade saw, they were polished using different grit sizes (from 600 to 1500) and examined under a microscope to ensure the appropriate sharpness of the edges of air voids. Afterwards, the surfaces of the samples were painted with a black marker with a broad nib to cover the prepared surface. The samples were then heated to 55 ${}^{\circ }$C and a white zinc paste was applied on the surface of the specimen. After cooling down, residual powder was removed from the surface of the specimen. Two specimens were tested for each case, and each sample was tested twice.

**Figure 4:**
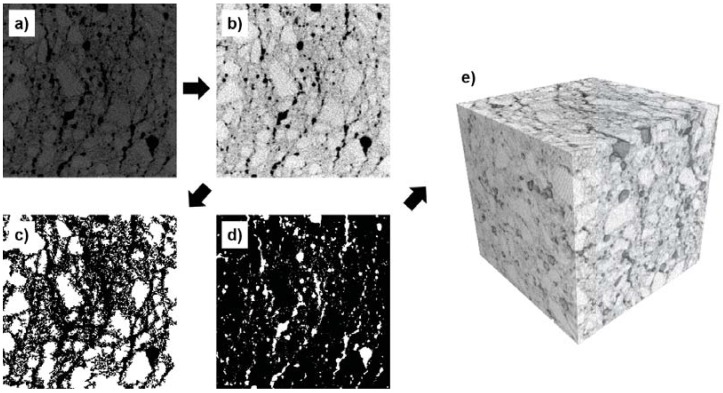
μ-CT imaging process to investigate pore and solid characteristics: (**a**) original μ-CT image; (**b**) contrasted image; (**c**) binarized image for aggregates; (**d**) binarized image for pores; (**e**) 3D μ-CT image (Note: in (**c**) and (**d**), the white regions represent aggregates (**c**) and pores (**d**) while the black is a background.).

To investigate the pore structures of the specimens in 3D, X-ray micro-CT (μ-CT) was also adopted. Figure 4 shows the μ-CT image processing used to classify aggregates and pores from the original image. In Figure 4a, the original 8-bit μ-CT image of the sample with crushed glass aggregates is presented. The original image is composed of 800×800 pixels which range from 0 (black) to 255 (white) with a pixel size of 29.7 μm. Median and contrast filters in MATLAB [36] were applied to the original image to enhance the image quality, as shown in Figure 4b. To classify specific components such as aggregates and pores, the filtered image was segmented using the multi-thresholding method [37] and the modified watershed algorithm [38]. Figure 4c,d are sample binary images of aggregates (crushed glass) and pores, respectively. In each figure, the white regions are aggregates (Figure 4c) and pores (Figure 4d), and black represents the background. A 3D image of the specimen was obtained by subsequent stacking of 2D images (Figure 4e). Using these segmented images, solid and pore characteristics including porosity can be effectively identified. Here, the microstructure of each specimen was classified into three phases: pore, aggregate, and solid phases. In general, the solid phase (binder) of concrete is composed of several components, i.e., calcium-silicate-hydrate (C-S-H) and calcium hydroxide (CH); however, the main objective of this study was to clarify the effect of different aggregates on concrete properties as well as their pore characteristics with the binder assumed to be a single phase for simplicity. In this study, we used the same cubic specimens which were used for the measurements of the thermal conductivity, and the core from the middle of each sample is used for μ-CT to obtain high-resolution images.

### 3.2. Thermal Conductivity Measurements

The thermal conductivity of the specimens was determined after curing the samples for 28 days. In order to ensure correct adhesion of the measuring probe, samples dried earlier were cut with a diamond saw in order to provide the proper flatness and parallelism of tested samples. Afterwards, thermal conductivity coefficients were registered. For thermal conductivity determination two apparatuses which used analysis of heat flow values in non-stationary conditions were used: Hot Disk (Göteborg, Sweden) and ISOMET (Nitra, Slovakia). These tools are a transient plate source method (Hot Disk) and a direct hand-held measuring instrument (ISOMET), which meet ISO (22007-2:2015) and European (EN 12571) standards, respectively. For the accuracy of the measurements, three specimens of each case were repeatedly tested with the mean value being presented.

### 3.3. Strength Measurements

The mechanical properties of the specimens were measured using sensitive experimental tools. Here, 28 days compressive and flexural strengths were evaluated to compare the mechanical performance of the lightweight concrete with different aggregates. To determine the strengths of the specimens, compression and flexural testing machines (Toni Technik, Berlin, Germany) were used according to EN 12390-3 and EN 12390-5, respectively. In addition, the dry densities of the specimens were measured at the age of 28 days. Three samples of each case were oven dried at 105 ${}^{\circ }$C until constant mass according to EN 12390-7, and the mean value was selected as the dry density. Detailed results of the material properties are shown and discussed in the following section.

## 4. Results and Discussions of the Effects of Different Aggregates

The material properties and characteristics of concrete specimens with three different aggregates were evaluated. For these purpose, the thermal (thermal conductivity) and mechanical (compressive and flexural strengths) of the specimens were measured. In particular, the pore characteristics of the specimens were investigated using different image-based approaches, such as SEM, μ-CT, and the RapidAir which is an automatic void analyzer.

### 4.1. Consistency and Material Density

The fresh properties of lightweight concrete are important in regulating material performance [39]. The consistency of the specimens was evaluated by conducting flow table tests (EN 12350-5) with the dosages of superplasticizer and stabilizer being adjusted to produce concrete with better consistency. Here, CG, EG, and NS specimens showed flow values between 550 and 650 mm (F5/F6) without segregation, which can be considered to be in the reasonable range. Although the surface of waste glass aggregate is much smoother and less impermeable than that of natural aggregate, much more superplasticizer is required to obtain similar initial concrete consistency. In the case of normal aggregates, 3.4 kg/m${}^{3}$ superplasticizer was added to get a flow diameter of 590 mm. In the case of crushed aggregates, the mix needed 8.75 kg/m${}^{3}$ of superplasticizer to achieve the same consistency as natural aggregates. This reflects the influence of the shape of crushed glass with the the angularity of the aggregate significantly affecting workability as a result of mechanical interlocking of the particles. Moreover, this effect was especially pronounced when the maximum size of aggregate used in concrete was limited to 4 mm because the shape of fine aggregates affects the workability of concretes much more than the geometrical properties of coarser aggregates [40].

However, in spite of the spherical shape and the very smooth surface of expanded glass (Poraver${}^{®}$), the mix needed more superplasticizer than normal aggregates. This can be explained by the differences in the densities between the normal aggregates and expanded aggregates. The heavy weight of normal aggregates increases the movement of particles during the workability test and consequently lower amount of superplasticizer is required.

The averaged material densities of the specimens were 1987 (CG), 634.4 (EG), and 2076.2 (NS) kg/m${}^{3}$; these results show that the use of crushed waste glass as an aggregate can reduce material density and meets the requirement for lightweight concrete (less than 2000 kg/m${}^{3}$) due to its lower specific gravity. The density of the EG specimen was significantly lower than that of other specimens since the expanded waste glass aggregates are porous material and have very low density. The characteristics of the aggregate types are presented later in Section 4.4.

### 4.2. Thermal Conductivity of the Specimens

**Figure 5:**
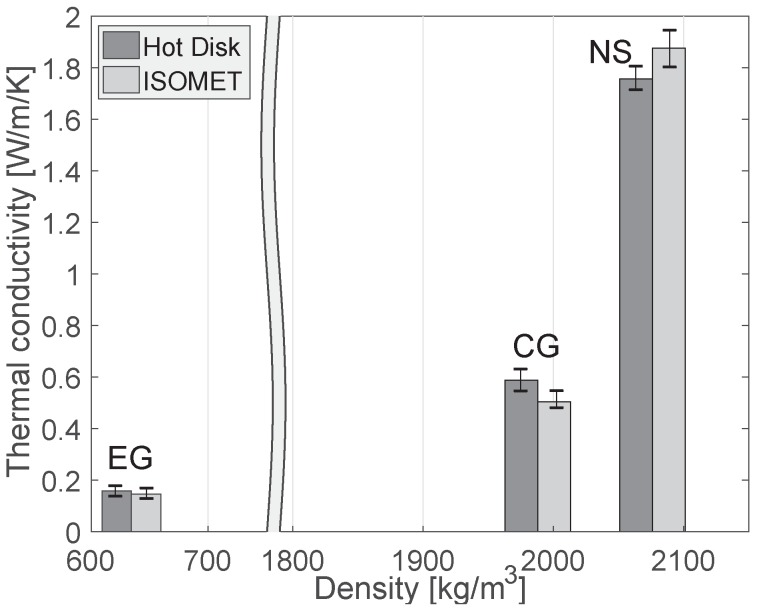
Thermal conductivity of the specimens measured with different devices.

The thermal conductivity of the specimens was measured using both the Hot Disk and ISOMET for more accuracy, and the results are presented in Figure 5. It can be seen that the differences between the two measurement tools are less than 7 %, thus being in reasonable agreement.

In Figure 5, the thermal conductivity of EG specimen is about 0.15 (W/m/K), being the lowest value of the specimens used in this study. As mentioned in the previous section, the EG specimen has very low density and porous components, and this strongly affected the insulation performance of the material. In addition, a significant decrement of the thermal conductivity of the CG specimen was observed compared with NS. Although the density difference between the CG and NS specimens was less than 100 kg/m${}^{3}$, the thermal conductivity of the concrete CG specimen was about one-third of NS, which can be considered a significantly lower thermal conductivity than that of normal concrete; this is mainly attributable to the low thermal conductivity of waste glass, which is consistent with the results in [41,42].

### 4.3. Mechanical Properties of the Specimens

**Figure 6:**
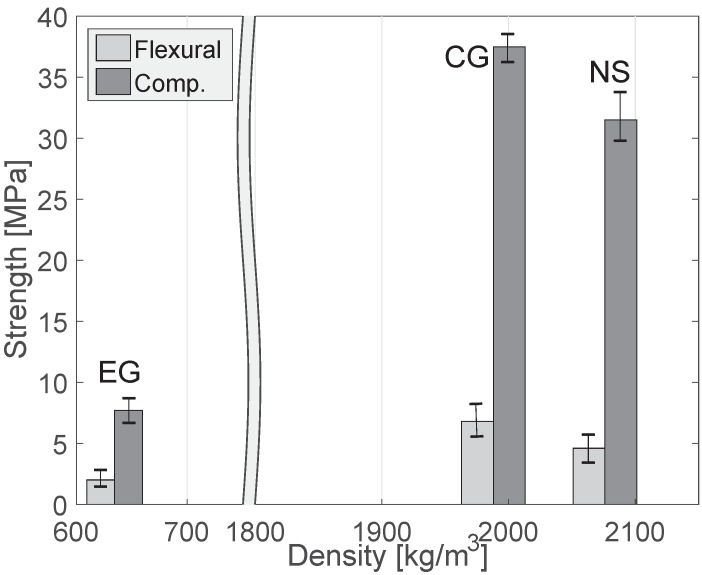
Compressive and flexural strengths of the specimens with different aggregates.

To investigate the mechanical performance of the specimens, both their flexural and compressive strengths after 28 days of curing are presented in Figure 6. The EG specimen presents the lowest flexural and compressive strength values from the samples in this study because of its low density and porous structure; for example, its compressive strength was about 20 % of that of the CG specimen. Figure 6 also shows that the replacement of natural sand aggregates with crushed glass aggregates results in an improvement in flexural and compressive strengths, although the density of the CG specimen is lower than that of the NS specimen. The effect of waste glass on the flexural strength of cement-based composites has been widely discussed in previous studies. For example, Du and Tan [43] have shown that higher amounts of finer crushed glass aggregate (75 wt % and 100 wt %) incorporated as a sand replacement in concrete can contribute to an improvement of flexural strength because of mechanical interlocking, internal friction, and increased aggregate surface area. Other studies have also shown that partial replacement of natural aggregate with glass aggregate can contribute to flexural strength improvement [41,44]. As can be seen in Figure 6, the results of this study in regard to flexural strength are consistent with those studies of flexural strength.

As with flexural strength, the specimen with crushed glass aggregates exhibited about 19% higher compressive strength than the specimen with natural sand aggregate. There are several studies which have reported deterioration with the use of waste glass for concrete [17,45,46], which is contrary to the results of the current study. For instance, it has been suggested in [35] that the durability of waste glass is much lower than that of natural aggregate when the size of glass particles is larger than 4.75 mm because of particle fractures. However, other researchers have reached results consistent with those of this study. For instance, Oliveira et al. [47] have observed that replacement of 100% of natural sand with glass sand (particle size < 4.76 mm) in concrete contributed to a 29% strength improvement compared with reference concrete. Du and Tan [43] and Taha and Nounu [48] have reported that 100% replacement of natural sand with glass sand with particle size < 4.75 mm [43] and 5 mm [48] shows little effect on the compressive strength of concretes after 28 days of curing, but that compressive strength tends to increase after 90 days; these studies used fine glass aggregates less than 4.7 mm particle size. In this study, only aggregates <4 mm were utilized for the specimens, and the obtained results show that the replacement of natural sand with crushed waste glass can enhance material strength. Therefore, it demonstrates that the particle size of glass aggregates affects the compressive strength of materials, and the use of finer aggregates less than 4.0 mm makes it possible to achieve an improvement in compressive strength.

### 4.4. Microstructures and Pore Characteristics

**Figure 7:**
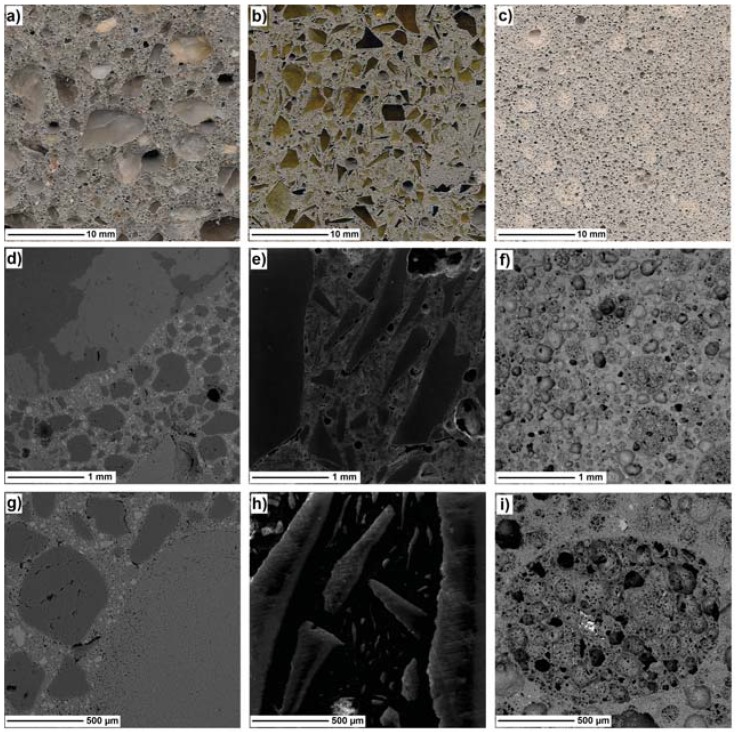
Scanned images of the sample surfaces: (**a**) NS; (**b**) CG; (**c**) EG; SEM micrographs: (**d**,**g**) NS; (**e**,**h**) CG; (**f**,**i**) EG.

Figure 7 presents scanned surfaces (a)–(c) and SEM micrographs (d)-(i) of the tested specimens. In these figures, it can be observed that the surfaces of NS (Figure 7a) and CG (Figure 7b) seem to be almost similar to dense aggregate particles, while the EG (Figure 7c) surface is much more porous than the other specimens. In Figure 7b,e, a large amount of flat, elongated, and needle-shaped glass particles can be observed in the CG specimen with even the finer particles of crushed glass <250 μm having angular and elongated shapes (Figure 7h); in our case, the presence of such geometrical characteristics in aggregates can attribute the mechanical interlocking of the particles as well as increased amounts of pores caused by the entrapping of air due to irregular shape [43]. In contrast, the NS specimen contains aggregates which have round-shaped particles (Figure 7d,g). Figure 7i presents the intersection of expanded glass aggregate in the concrete matrix. This figure shows that the aggregates are highly porous contributing to the significant increment of concrete porosity and significant decrement of the mechanical properties of the EG specimen.

**Figure 8:**
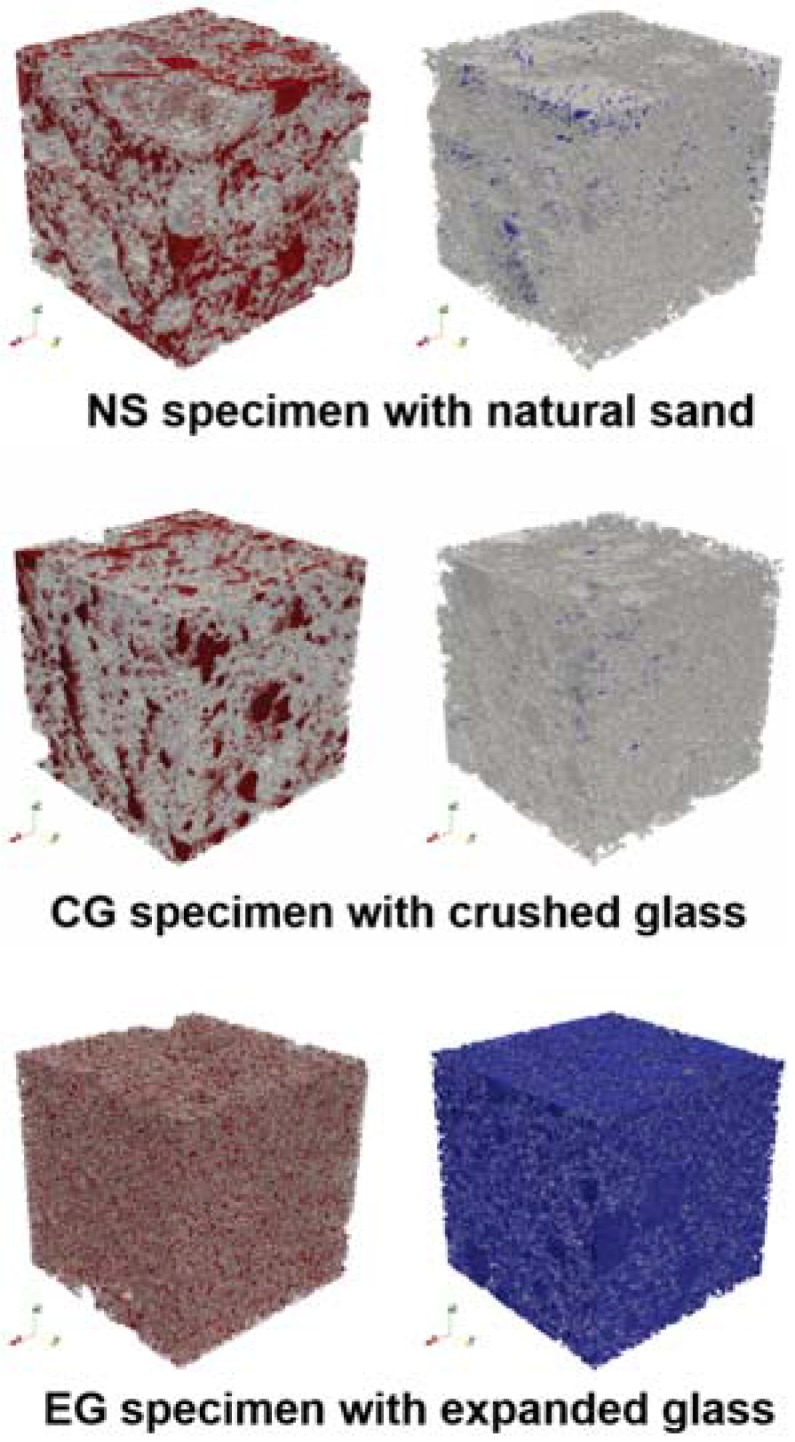
Pore structures of the specimens with different aggregates (Note: in each sub-figure, the left figure presents the pores within the solid (matrix) (red), while the right figure presents the pores inside aggregate particles (blue).).

**Figure 9:**
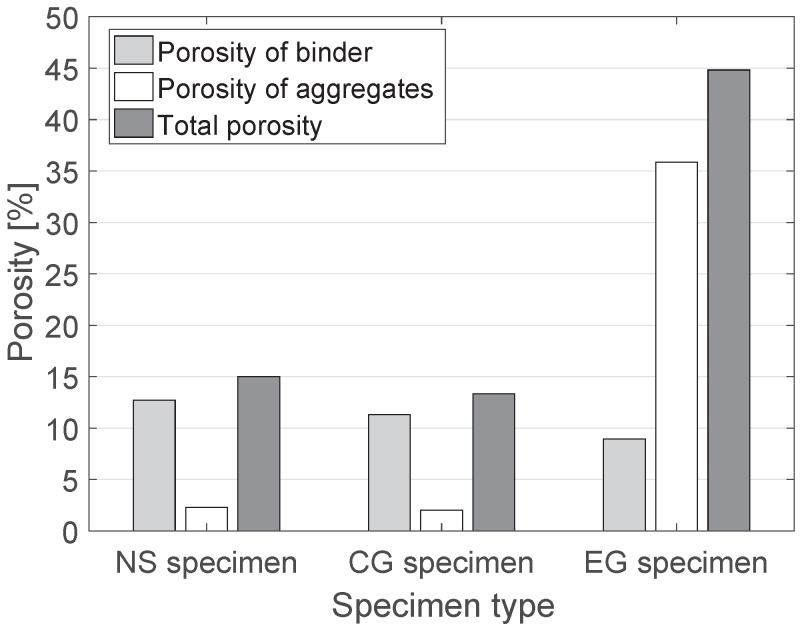
Porosity data of each phase in the specimens (Note: the total porosity values of each case are 15.1%, 13.4%, and 44.8% for NS, CG, and EG specimens, respectively.).

For more detailed investigation of the pore structures of the specimens, μ-CT images were utilized. Figure 8 shows segmented pore distributions of the specimens in 3D. The pore structures and porosity data of the specimens are presented in Figure 8 and Figure 9. According to the μ-CT images, a general trend of the pore structures can be identified. In Figure 8, the left figure presents the pores (red) in the binder, and the blue region of the right figure presents the pores inside aggregates. The gray-colored phases denote the binder (left) and the aggregates (right) in each case. For NS and CG specimens in Figure 8, most of the pores are located in the binder region, and only a small amount of pores can be found inside the aggregates; this denotes that both natural sand and crushed waste glass are dense materials, and that material properties related to porosity can be strongly affected by the pores in the binder phase. In contrast, the aggregates of the EG specimen contain numerous pores inside the particles, which can affect the mechanical and thermal properties of materials.

In addition, the pore distribution of the binder of EG is more dispersed than that of the NS and EG specimens. From the figure, the effect of the aggregates on the material characteristics can be predicted; the aggregate type affects both the porosity and the pore distribution of concrete materials.

The detailed porosity of the specimens was calculated using the images in Figure 8. In Figure 9, the porosity values in each phase of the specimens are presented, and the pore characteristics discussed above are quantitatively confirmed. As shown in the μ-CT images, the porosity of the EG specimen is significantly larger than that of the other specimens; this result being mainly from the highly porous aggregates in the EG specimen. It should also be mentioned that the porosity of natural sand aggregates is slightly larger than that of crushed waste glass aggregates, and this affects the difference between the characteristics of specimens with each aggregate.

**Figure 10:**
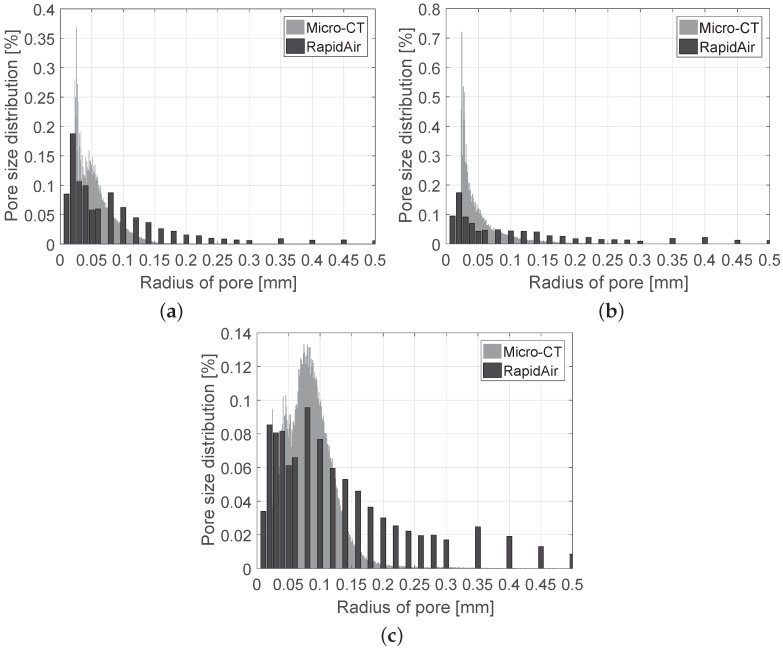
Pore size distributions of the specimens measured from μ-CT and RapidAir: (**a**) NS specimen; (**b**) CG specimen; (**c**) EG specimen.

The pore size distributions of each specimen were also investigated using the μ-CT images and the RapidAir device. The porosity values measured with the RapidAir were 15.9% (NS), 13.1% (CG), and 46.7% (EG), which are almost the same as those from the μ-CT images. Figure 10 shows the pore size distributions of the specimens. In all cases, the general trend of the pore size distribution is almost identical, even though the peak values and the distribution of the pores larger than 300 μm are slightly different; the differences are attributable to the heterogeneity of the specimens because the RapidAir is a method based on a 2D image, while the μ-CT is a 3D based method. In this figure, the EG specimen includes more pores than other specimens, and pores >100 μm are dominant in this specimen. Moreover, compared to the NS specimen, the CG specimen mainly contains pores <60 μm, which are affected by the interlocking of crushed glass and can lead to larger mechanical properties as well as smaller thermal conductivity of the material.

From the results of the material properties and the pore characteristics, it is concluded that the use of crushed waste glass aggregates can reduce concrete density and thermal conductivity significantly with the beneficial effect of enhancing compressive and flexural strengths. The results indicate that aggregate characteristics, such as shape, pore size, porosity, and material properties, strongly affect concrete materials, and materials with advanced mechanical properties as well as insulation can be achieved by using crushed waste glass as a lightweight aggregate.

## 5. Conclusions

Lightweight concrete specimens with different sources of aggregate were produced and investigated in this study. In particular, the effect of recycled crushed waste glass on concrete was examined according to several investigative approaches. To produce lightweight concrete specimens, crushed waste glass, natural sand, and expanded glass were adopted. Aggregate grading was also considered in maximizing the aggregate content. Pore and solid characteristics of specimens were evaluated using SEM, μ-CT, and RapidAir, which is an automated image analysis tool. Both the flexural and compressive strengths of the materials were measured using sensitive experimental devices, and thermal conductivity was also evaluated using a Hot Disk and a ISOMET in order to enhance accuracy.

The concluding remarks of this paper can be summarized as follows:

- Lightweight concrete of density less than 2000 kg/m${}^{3}$ can be produced by using crushed waste glass aggregates. Specimens using crushed waste glass show a compressive strength above 36 (MPa) and a thermal conductivity less than 0.6 (W/m/K), and this can be considered an effective lightweight concrete which satisfies both mechanical and thermal properties.
- The image-based methods adopted here, such as SEM and μ-CT imaging, were effectively utilized to investigate the microstructures of the specimens from different perspectives. Particle shape and the intersection between binder and aggregate can be examined using SEM, and pore characteristics, such as the porosity of each component and the pore size, can be nondestructively evaluated using μ-CT.
- The shape of crushed glass aggregates affects the fresh properties of the material. The angularity of the aggregates influences workability due to mechanical interlocking of the glass particles resulting in a need for more superplasticizer and stabilizer to achieve appropriate concrete consistency.
- The specimen with crushed waste glass had about 20 % more compressive strength than the specimen with natural sand, with the thermal conductivity being less than one-third of normal concrete. Therefore, the use of crushed glass can enhance material properties significantly.
- When a material contains dense aggregates, such as crushed glass and natural sand, the porosity of the specimen is mainly determined by the binder phase. However, for a material with porous aggregates, e.g., expanded glass, the porosity of the aggregates is the dominant factor regulating material properties.

In addition to the work reported in this paper, more parametric studies with different mix proportions using crushed waste glass are required to clarify the effects of recycled material on concrete properties.

## Figures and Tables

**Table 1:** Chemical and physical properties of cement and silica fume given by the manufacturer (wt %).

Material	CaO	SiO${}_{2}$	Al${}_{2}$O${}_{3}$	Fe${}_{2}$O${}_{3}$	MgO	Na${}_{2}$O	K${}_{2}$O	SO${}_{3}$	Specific Density	Surface Area (cm${}^{2}$/g)
CEM I 42.5 N	63.14	20.53	5.33	2.36	1.49	0.21	0.72	3.39	3.05	3860
Silica fume	0.2	98.4	0.2	0.01	0.1	0.15	0.2	0.1	2.2	200,000

**Table 2:** Physical properties of aggregates.

Material	Natural Sand		Crushed Glass	Expanded Glass (Poraver${}^{®}$)				
**Particle size (mm)**	**0–2**	**2–4**	**0–4**	≤**0.125**	**0.125–0.250**	**0.50–1**	**1–2**	**2–4**
**Specific density**	2.61	2.63	2.53	0.85	0.70	0.50	0.40	0.32
**Water absorption (wt %)**	0.60	0.30	0.20	28	28	20	20	23

**Table 3:** Mix proportions and properties of fresh concrete (kg/m${}^{3}$).

Specimen	Cement	SF *	Water (w/b=0.6) ***	Natural Sand (0–4 mm)	Crushed Glass (0–4 mm)	Expanded Glass (0–4 mm)	SP **	Fresh Density	Flow Diameter [mm]
**NS**	306	34	204	1673	-	-	3.4	2260	590
**CG**	306	34	204	-	1610	-	8.75	2080	590
**EG**	306	34	204	-	-	342	6.12	872	550

* SF: silica fume, ** SP: superplasticizer, *** the water absorbed by aggregate is additionally added during the mixing process.
